# Do poor people in the poorer states pay more for healthcare in India?

**DOI:** 10.1186/s12889-019-7342-8

**Published:** 2019-07-30

**Authors:** Anjali Dash, Sanjay K. Mohanty

**Affiliations:** 1 International Institute for Population Sciences, Govandi Station Road, Deonar, Mumbai, Maharashtra 400088 India; 2 Department of Fertility Studies, International Institute for Population Sciences, Govandi Station Road, Deonar, Mumbai, 400088 India

**Keywords:** Public health, Hospitalization, Out-of-pocket expenditure, Financial protection, Poverty, India

## Abstract

**Background:**

Rising health spending is associated with high out-of-pocket expenditure (OOPE), catastrophic health spending (CHS), increasing poverty, and impoverishment. Though studies have examined poverty and impoverishment effect of health spending in India, there is limited research on the regional patterns of health spending by type of health centers. This paper tests the hypothesis that the poor people from the poorer states of India pay significantly more for hospitalization in public health centers than those in the richer states of India.

**Methods:**

Data from the Social Consumption of Health Survey (71st round, 2014), carried out by the National Sample Survey (NSS) is used in the analyses. Descriptive statistics, log-linear regression model and tobit model were used to examine the determinants and variations in health spending.

**Results:**

Inter-state variations in the utilization of public health services and the OOPE on hospitalization are high in India. States with high levels of poverty make higher use of the public health centers and yet incur high OOPE. In 2014, the mean OOPE per episode of hospitalization in public health centers in India was ₹5688 and ₹4264 for the economically poor households. It was lowest in the economically developed state of Tamil Nadu and highest in the economically poorer state of Bihar. The OOPE per episode of hospitalization in public health centers among the poor in the poorer states was at least twice that in Tamil Nadu. Among the poor using public health centers, the share of direct cost account 24% in Tamil Nadu compared to over 80% in Bihar, Odisha and other poorer states. Adjusting for socio-economic correlates, the cost of hospitalization per episode (CHPE) among the poor using public health centers was 51% lower than for the non-poor using private health centers in India.

**Conclusion:**

The poor people in the poorer states in India pay significantly more to avail hospitalization in public health centers than those in the developed states. Provision of free medicines, surgery and free diagnostic tests in public health centers may reduce the high OOPE and medical poverty in India.

**Electronic supplementary material:**

The online version of this article (10.1186/s12889-019-7342-8) contains supplementary material, which is available to authorized users.

## Background

Over a hundred million people are pushed into poverty annually due to health spending, and the impact of health spending on poverty is high in low-resource settings [1–5]. About 97 million people were impoverished due to spending on health care in 2010 [1]. Globally, an estimated 588 million (9.7%) people had incurred CHS in 2000 and it has increased to 808 million (11.7%) in 2010 [5]. Evidence from 14 low and middle income countries of Asia suggests that poorer countries, with low social protection, rely heavily on direct payments and the poverty impact of health spending is high [1, 3]. In 2014, the OOPE on health, defined as health expenditure net of reimbursement, was the highest in Comoros (76%), followed by Yemen and lowest (less than 1%) in New Zealand followed by Kiribati [2]. High OOPE is associated with increasing non-communicable diseases (NCDs), increase in the share and the size of the elderly population, increasing cost of health care, increasing income levels, low coverage of health insurance, advancements in medical technology and low public spending on health care [6–9]. Besides, the opportunity cost of medical care is very high in developing countries [10–14]. High expenditure on health adds to unequal access in health care utilization across regions and socio-economic groups [15, 16]. Affordable health services are a fundamental goal of welfare states, and protecting households from CHS is a key monitoring indicator of the sustainable development goals (SDGs) [2, 17, 18].

The high OOPE on health care affects the poor and the vulnerable the most, and has drawn considerable attention from researchers and policy makers in developing countries. Protecting people from increasing health care costs is also a priority developmnet agenda worldwide. However, a sizeable proportion of the population does not seek health care due to increasing health care costs. The high OOPE in developing countries leads to a reduction in the consumption of non-food goods [19], and increases the incidence of untreated morbidity, particularly among the rural, poor, female-headed and elderly households [6, 20]. Studies suggests that medical poverty is high among households with chronic NCDs, and due to high share of medicines on total cost of hospitalization [21–24]. Owing to heavy reliance on direct OOPE and low financial protection by government health systems [1, 4] the poor often deprived of access to health care [25–27]. Since the Alma-Ata declaration in 1978, there has been a global effort to provide accessible and affordable quality of health care services through universal health coverage (UHC) [28]. Evidence shows that UHC has helped reduce financial hardships in many developing countries but not progressive in India [29, 30].

### The Indian scenario

India’s health system is characterized by the co-existence of public and private health care providers. Public health facilities are provided by the central government, the state governments, and local bodies [17, 31]. Public health centers provide low cost care, are generally overcrowded, and largely used by the poor. Despite the low cost of health care in public health facilities, the poor households incur a high CHS and bear a higher burden of diseases [31–34]. About 71.1% of the health spending in 2004 and 67.74% in 2014 was met by households [35]^.^ Public health spending in India was about 1% of gross domestic product (GDP), lower than that in many low income countries. Evidence shows that medical poverty owing to high OOPE increased from 32.5 million in 1999–2000 to 50.6 million in 2011–12 [25, 36–38]. Every year, 3.5 to 6.2% of the population of India was pushed into poverty due to high OOPE [3, 25, 39]. The impoverishment effect of hospitalization cases is also high [16, 40]. Similarly, about 23.4% households incurred CHS during 2011–12 [27]. The national average in health spending conceals large variations across the states of India. The high burden of health spending in India affects the poorer states, the poorer regions/districts within the states, and the poor considerably more than the richer counterparts [21, 40–42]. Evidence shows that despite an increase in insurance coverage financial risk protection has not been reduced [43–45].

Earlier studies in India found large inter-state variations in the per capita health spending, OOPE, CHS and impoverishment effect of health spending across states, residence, gender, and economic groups [27, 41, 46, 47]. Some empirical studies focused on expenditure on out-patient by type of health care providers [9, 48]. Though studies have examined on the disparities in health outcomes, access, coverage, and quality of care [17, 49–51], there are limited number of studies on inter-state variations in health spending in India [41, 52]. Moreover, none of the studies have focused on inter-state variation in health spending with respect to type of health centers and poverty. In this context the main objective of this paper is to examine the OOPE on hospitalization in public and private health centers by level of poverty across the states of India. It seeks to answer the following research questions: Do poor people in the poorer states pay more for hospitalization in public health centers? What are the factors leading to high payments at public health centers in the poorer states? It tests the hypothesis that the poor from the poorer states of India pay significantly more on hospitalization in public health centers than the poor in the developed states in India.

## Methods

### Data

This study used unit data of Social Consumption on Health, schedule 25.0 of the 71st round (2014), collected by the NSS, Government of India from January to June 2014. The NSS, under the Ministry of Statistics and Program Implementation, has been conducting nationwide large sample surveys since its inception in 1950. It collected information on a wide range of socio-economic issues such as consumption, employment and unemployment, morbidity and health care, education, migration etc. in its various rounds. The first comprehensive morbidity and health care survey was conducted in the 42nd round, July 1986–June 1987. Since then, three rounds of health surveys (52nd, 60th and 71st) have been conducted. The instruments and sampling coverage have been revised over time. The 60th round conducted in 2004 and the 71st round conducted in 2014 are the latest two rounds of health surveys. Details methodology and findings of the 71st round are available in the report [53]. Owing to increasing demand, the NSS conducted a health survey in its 75th round (July 2017–June 2018) and the data/report is yet to be released. The 71st round unit level data is available from Indian Council of Social Science Research (ICSSR) data repository and could be accessed upon a data request through http://www.icssrdataservice.in/datarepository/index.php/catalog/107.

The 71st round survey provided comprehensive information on the type of treatment, disease pattern, type of health centers (public and private), the amount reimbursed, source of financing, etc. based on a 365 day reference period of hospitalization and a 15 day reference period for out-patient care. In this study, we have used episode of hospitalization in a 365 day reference period as the unit of analyses. The survey covered all 36 states, including the union territories of India. A total of 65,932 households (335,499 individuals of which 333,104 survived and 2,395 death cases) were successfully interviewed. We retain the survival and death cases for the analysis, as health spending on individuals who eventually died was significantly higher than that on individuals who survived [54].

To understand the supply side factors of the health sector, we have used the government reports published by the Ministry of Health and Family Welfare (MoHFW), Government of India and the 4th round of District Level Households and Facility Survey (DLHS-4). The DLHS-4 was carried out by the International Institute for Population Sciences (IIPS) during 2012–13. It was the first ever survey in the Indian context that provides information on supply side variables. The district level estimates are available for public use and unit data could be accessed upon request through http://rchiips.org/DLHS-4.html.The survey covered in four tiers of health facilities: sub center (SC), primary health center (PHC), community health center (CHC) and district hospital (DH). The survey provided information on human resources (number of doctors and paramedical staffs), accessibility of health facilities (distance from villages to PHC) and related information at the facility level [55]. We have used the following variables at the state level: shortfall of public health centers such as SC, PHC, CHC, DH, shortfall of a medical doctor, shortfall of paramedical staffs, shortfall of bed, density of bed per one lakh population and health workers per 1000 population At the district level, we have computed the shortfall of doctor, nurse and bed. We prefer to use the shortfall in health infrastructure, manpower and distance at the PHC level due to data constraints. Though information on district hospitals was available in DLHS-4, there was one district hospital per district and hence no variation could be shown.

In the present study, we focused on 19 major states along with Delhi, where the number of hospitalized cases was 300 and more in each public and private health care center. We have used state-specific poverty lines by rural and urban areas, as suggested by the Rangarajan committee to identify the poor and the non-poor [56]. The state specific poverty line is a standard measure of comparison of the poverty level and the poverty cut-off is higher in richer states than in the poorer states. Tamil Nadu was used as a reference state as it had low OOPE on hospitalization in public health centers.

### Outcome variable

A composite variable combining type of health centers (public and private) and poverty (poor and non-poor) was computed and categorized into four distinct groups. These are individuals belonging to (i) poor households and using public health centers (ii) poor households and using private health centers (iii) non-poor households and using the public health centers and (iv) non-poor households and using private health centers. The OOPE on hospitalization was computed for each of these four categories across the states of India. Cost of hospitalization per episode (CHPE) is used as an outcome variable in multivariate analyses and reimbursement was used as an independent variable in the regression model.

### Independent variables

The covariates used in the analysis were: residence (rural, urban), religion (Hindu, Muslim, Others), caste (schedule caste/schedule tribe (SC/ST), other backward classes (OBC), others), sex (male, female), poverty and health care utilization (poor using public health centers, poor using private health centers, non-poor using public health centers, non-poor using private health centers), age (<=14, 15–59, > = 60), surgery (not received, free or partly free, on payment), medicine (not received, free or partly free, on payment), x-ray (not received, free or partly free, on payment), diagnostic (not received, free or partly free, on payment), and reimbursement of health expenditure (no insurance, insurance but did not benefit, benefited from insurance), disease (cancer, bone disease, diabetes, fever, high blood pressure, accident, jaundice/respiratory, heart, eye, tuberculosis, blood disease, neurological, others).

#### Statistical analysis

Descriptive statistics, bivariate and multivariate analyses were used. Log-linear regression model and Tobit regression model were used in this analysis. Log linear regression model used as CHPE was a continuous variable and skewed. The general regression model used for the study is defined in eq. 1.

1 $$ {\displaystyle \begin{array}{l} Ln(CHPE)=\alpha +{\beta}_1\ {res}_i+{\beta}_2\ {age}_i+{\beta}_3\ {sex}_i+{\beta}_4\ {religion}_i+{\beta}_5\ {caste}_i+{\beta}_6\  pov\_{hos}_i+{\beta}_7\ {medicine}_i+{\beta}_8\ {surgery}_i+\\ {}\kern2.52em {\beta}_9\;{xray}_i+{\beta}_{10}\;{diagnostic}_i+{\beta}_{11}\;{insurance}_i+{\beta}_{12}\;{diseases}_i+{e}_i\end{array}} $$

where α is the intercept, subscript i is used for episode of hospitalization, res is residence, age is age of patients, pov_hos is composite variable of poverty and type of health centers, medicine is the services of medicine, surgery is the services of surgery, x-ray is the services of x-ray, diagnostic is the services of diagnostic tests, insurance is the benefit or status of insurance, and diseases is the broad disease classification. The analyses are conducted by using sampling weights to secure representativeness of the estimation.

### Robustness analysis

We have used the Tobit model as an alternate model to check the robustness of the main results. The Model specification remains the same as of eq. 1.

The Tobit model is defined as:

2 $$ {\mathrm{CHPE}}_{\mathrm{i}}={\upbeta}_{\mathrm{i}}{{\mathrm{X}}_{\mathrm{i}}}^{'}+{\mathrm{e}}_{\mathrm{i}} $$

where, CHPE_i_ is the cost of hospitalization per episode in Indian rupees (₹) and X_i_^’^ is vectors of independent variables. The set of independent variables are similar as of eq. 1. β_i_ is the regression coefficient, and e_i_ error terms. Results are presented as marginal effects and estimates are provided for India, Bihar and Tamil Nadu.

## Results

Table 1 presents the descriptive statistics of the sample households and individuals covered in the survey. Among all households surveyed, 12% of the households were poor and using public health centers, 6% were poor and using private health centers, 19% were non-poor and using public health centers and 23% were non-poor and using private health centers and 41% did not seek hospitalization care. About 60% of the households, one member or more was hospitalized. In 2014, the mean age of hospitalization was 34 years, the monthly per capita expenditure (MPCE) of the households was ₹1625, and it was the lowest among the poorer households who were hospitalized in public health centers (₹807) and the highest among the non-poor households who were hospitalized in private health centers (₹2245).

**Table 1:** Monthly per capita consumption expenditure, mean age and percentage urban by poverty and type of health centers in India, 2014

Parameters	Poor using public health centers	Poor using private health centers	Non-poor using public health centers	Non-poor using private health centers	India
Total Number of household surveyed	7,642	4,051	12,343	14,939	65932
Total number of hospitalized case	10,033	6,278	17,406	23,739	57,456
% residing in urban areas^a^	23.97	35.12	20.68	37.46	29.98
Mean age^a^	31	32	34	37	34
MPCE (in Indian rupees)^a^	807	880	1654	2245	1625

^a^t-test shows significant differences in mean age and MPCE of poor using public health centers and each of the three other groups. Chi-square test shows significant differences in percentage urban across groups

### Inter-state variations in the utilization of public and private health centers for hospitalization care

**Fig. 1:**
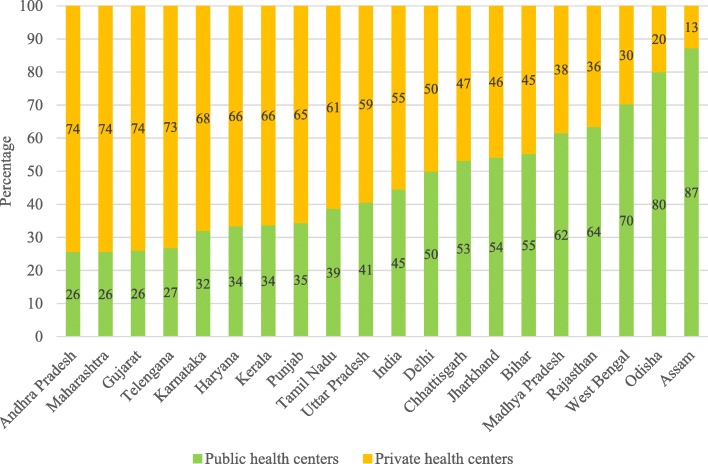
Percent distribution of hospitalization in public and private health centers in major states of India, 2014

Figure 1 presents the percentage distribution of hospitalization in public and private health centers in the states of India. The utilization of health services in public health centers was the highest in Assam (87%), followed by Odisha (80%), West Bengal (70%), Rajasthan (64%), Madhya Pradesh (62%), and Bihar (55%). On the other hand, the utilization of health services in private health centers was 74% each in Andhra Pradesh, Maharashtra and Gujarat. Barring Uttar Pradesh and Andhra Pradesh, states that were economically better-off used more of the private health centers, while poorer states used more of the public health centers.

Appendix 1 of the Additional file 1 presents hospitalization by disease and type of health centers across the states of India. Among those hospitalized in public health centers, about 45% each were hospitalized for NCDs and communicable diseases (CDs). In the case of private health centers, two-thirds of the hospitalized cases were for NCDs and about one-fifth of the cases were for CDs. The state pattern suggests that the majority of the hospitalized cases in private health centers were for NCDs. In Kerala, about 68% of the hospitalized cases in public health centers were for NCDs while it was 21% in Jharkhand followed by 23% in Bihar. This pattern was opposite in the case of CDs. More than half of the hospitalization cases in private health centers across all the states was for NCDs.

### Poverty and hospitalization in the states of India

**Fig. 2:**
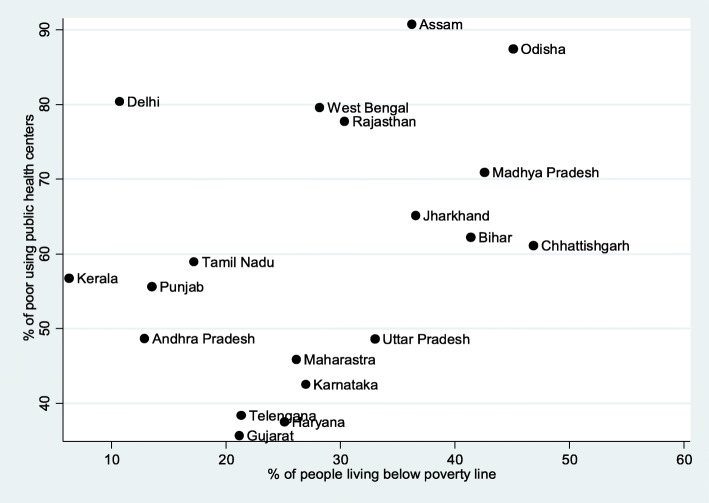
Percentage of population living below poverty line and hospitalization in public health centers in major states of India (pdf 437 kb)

Figure 2 presents the scatter plot of the percentage of population living below the poverty line and the percentage of the poor hospitalized in public health centers. Among the 19 states of India, the poverty level was over 40% in Chhattisgarh, Odisha, Madhya Pradesh, and Bihar and less than 15% in the states of Kerala, Delhi, Punjab, and Andhra Pradesh. In general, the poor belonging to the states with a higher poverty level were using more health services from the public health centers for hospitalization care.

Table 2 presents the utilization of public health centers among the poor and non-poor in states of India. The use of public health centers was higher among poor than non-poor in each of the state of India. In India, about 61% of the poor were using public health centers compared to 40% of the non-poor. The proportion of poor utilizing public health centers for hospitalization was highest in Assam followed by Odisha, Delhi, West Bengal and Rajasthan. It was low in Gujarat, Haryana, and Telangana.

**Table 2:** Hospitalization (%) among the poor and non-poor in public and private health centers of India, 2014

States	% of poor hospitalized in public health centers	% of non-poor hospitalized in public health centers	T statistic	% of people living below poverty line	Number of episode of hospitalization		
					Poor	Non-poor	All
Tamil Nadu	58.90	34.70	11.15	17.22	671	3118	3789
Jharkhand	65.13	48.03	6.21	36.57	432	758	1190
Telengana	38.35	23.92	6.81	21.34	257	1018	1275
Andhra Pradesh	48.66	22.45	10.05	12.91	368	2021	2389
Rajasthan	77.76	57.42	8.88	30.37	742	1888	2630
Kerala	56.76	32.31	9.17	6.31	257	2745	3002
Chhattisgarh	61.12	46.53	5.79	46.87	408	492	900
Karnataka	42.53	28.38	7.30	26.97	773	2059	2832
Maharashtra	45.89	18.77	17.35	26.16	1471	3538	5009
Madhya Pradesh	70.90	54.90	11.62	42.59	1292	1820	3112
Bihar	62.24	50.56	4.41	41.40	1016	1359	2375
Gujarat	35.71	23.67	5.99	21.17	594	2105	2699
Assam	90.71	85.50	5.23	36.24	619	1091	1710
Odisha	87.43	74.12	10.01	45.09	953	1159	2112
West Bengal	79.57	66.82	12.70	28.19	1318	3173	4491
Uttar Pradesh	48.60	36.79	7.84	33.02	2195	4244	6439
Haryana	37.51	32.21	3.84	25.10	331	942	1273
Delhi	83.10	46.27	5.52	10.70	85	785	870
Punjab	55.64	31.19	5.44	13.54	182	1146	1328
India	60.71	39.80	42.20	26.80	27439	30017	57456

**Fig. 3:**
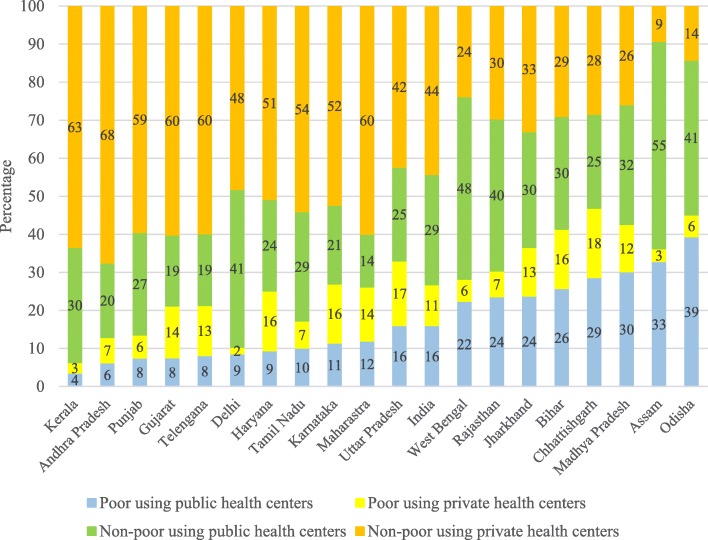
Percentage distribution of poor and non-poor hospitalized in public and private health centers in states of India, 2014

Figure 3 presents the inter-state variations in hospitalization by public and private health centers and poverty level in India. Among those hospitalized, 16% belonged to poor households and used public health centers, 11% were poor households and used private health centers, 29% were non-poor households using public health centers and 44% were non-poor households using private health centers. The state pattern is striking; a higher proportion of the poor were using public health centers in the poorer states of Odisha (39%), Assam (33%), Madhya Pradesh (30%), Chhattisgarh (29%) and Bihar (26%). In many of the poorer states, a significantly higher proportion of population was using public health centers. It also suggests that a higher proportion of the non-poor in the economically better-off states utilized private health centers.

### Inter-state variations in OOPE on hospitalization in public and private health centers by level of poverty

Table 3 presents the OOPE among the poor and the non-poor by type of health care centers in the states of India. In 2014, the mean OOPE per episode of hospitalization in public health centers was ₹5688 compared to ₹24924 in private health centers. The OOPE per episode of hospitalization in public health centers was lowest in Tamil Nadu (₹2395) and highest in Punjab (₹10540). On the other hand, the OOPE per episode of hospitalization among the poor in public health centers of the empowered action group states (EAGs), namely, Bihar, Jharkhand, Odisha, Madhya Pradesh, Chhattisgarh, Assam, and Uttar Pradesh was higher than that in Tamil Nadu. The OOPE per episode of hospitalization of the poor using private health centers was consistently lower than that of the non-poor using private health centers across the states of India. Among the states the non-poor using private health centers, the OOPE was highest in Delhi (₹44208) and lowest in Jharkhand (₹15835). The OOPE was higher in private health centers than in public health centers in each state of India. The coefficient of variation of OOPE in public health centers was 35 and that in private health centers was 28. In most of the states, the OOPE accounts for more than 95% of total health expenditure of hospitalization care.

**Table 3:** Out-of-pocket expenditure (₹) per episode of hospitalization by poverty and type of health centers in the states of India, 2014

States	Poor using public health centers	Poor using private health centers	Non-poor using public health centers	Non-poor using private health centers	All	F-statistics	Public health centers	Private health centers	OOPE as a share of Total cost
Tamil Nadu	1900	17485	2569	29444	18084	145.80	2395	28059	93.87
Jharkhand	2534	15638	4972	15835	9333	21.03	3903	15780	98.83
Telangana	2696	19328	4715	28136	20488	12.20	4103	26549	98.20
Andhra Pradesh	2477	11353	4881	24841	18640	25.60	4296	23636	98.63
Rajasthan	2790	11946	5191	26875	11510	119.21	4299	24105	96.41
Kerala	3863	19222	4392	24559	17568	45.94	4336	24339	96.02
Chhattisgarh	2881	18072	6371	27186	13416	27.59	4498	23625	98.06
Karnataka	4080	16231	4887	22114	15563	62.16	4600	20769	91.39
Maharashtra	3701	22381	5398	27071	20598	72.09	4610	26176	91.60
Madhya Pradesh	3190	21372	6614	26675	12604	91.50	4939	24959	91.29
Bihar	4128	17359	6631	20730	11748	62.79	5467	19549	98.56
Gujarat	2347	11935	7037	18461	14223	31.92	5684	17257	92.83
Assam	5557	13953	6907	54097	11064	60.52	6399	43381	96.96
Odisha	5882	25973	7481	35055	11818	105.61	6694	32466	97.81
West Bengal	7930	23462	6840	31778	13983	107.64	7187	30159	92.40
Uttar Pradesh	3085	17143	9981	29412	18317	109.26	7262	25902	99.02
Haryana	6840	28127	8931	24870	19838	22.84	8344	25639	89.62
Delhi	4218	66844	9644	44208	26829	19.17	8713	45000	85.27
Punjab	8715	22989	11050	33974	25229	8.44	10540	32966	96.49
India	4264	18454	6482	26470	16189	1106.11	5688	24924	94.82

Note: F statistics shows significance differences (***p < 0.001) among groups in each of the state of India

Appendix 2 of Additional file 2 presents the mean OOPE per episode of hospitalization by type of disease and health centers in the states of India. The mean OOPE per episode of hospitalization for NCDs in public health centers was ₹7485 compared to ₹24927 in private health centers of India. Among the four broad diseases categories, the OOPE per episode of hospitalization for accident and others was highest in both public and private health centers. The state pattern was similar but there were large variations among the states. In Tamil Nadu, the mean OOPE per episode of hospitalization for treating NCDs in public health centers was ₹2187 compared to ₹26008 in private health centers. It was highest in the poorer states of Uttar Pradesh (₹16406), followed by Bihar (₹ 10796). The mean OOPE per episode of hospitalization in private health centers for NCDs was highest in Delhi (₹47187) and lowest in Jharkhand (₹16364). In case of CDs hospitalized in public health centers, it was highest in Assam (₹5101) followed by Odisha (₹4800) and lowest in Gujarat (₹1427).

Table 4 presents the ratio of OOPE on hospitalization by type of health center and extent of poverty in states of India. The OOPE of Tamil Nadu has been taken as the reference category. The OOPE for hospitalization in public health centers of India was 2.38 times higher than that in Tamil Nadu (column 7, Table 4). The OOPE in public health centers in the poorer states of Bihar, Odisha, and Uttar Pradesh was higher than that in Tamil Nadu by 2.3, 2.8 and 3.0 times respectively. The OOPE of the poor using public health centers in the poorer states of Odisha was 3.10 times higher than those in Tamil Nadu. Inter-state variations were not large in the private health centers of India. The ratio of non-poor using private health centers was lower in Jharkhand (0.54) than in Tamil Nadu.

**Table 4:** Ratio of out-of-pocket expenditure on hospitalization among major states of India with respect to Tamil Nadu, 2014

States	Poor using public health centers	Poor using private health centers	Non-poor using public health centers	Non-poor using private health centers	All	Public health centers	Private health centers
Tamil Nadu	1.00	1.00	1.00	1.00	1.00	1.00	1.00
Jharkhand	1.33***	0.89	1.94	0.54***	0.52***	1.63***	0.56***
Telangana	1.42***	1.11	1.84***	0.96	1.13***	1.71***	0.95
Andhra Pradesh	1.30	0.65***	1.90***	0.84	1.03	1.79***	0.84***
Rajasthan	1.47***	0.68**	2.02***	0.91	0.64***	1.80***	0.86**
Kerala	2.03***	1.10	1.71***	0.83***	0.97	1.81***	0.87***
Chhattisgarh	1.52**	1.03	2.48***	0.92	0.74	1.88***	0.84
Karnataka	2.15***	0.93	1.90***	0.75***	0.86	1.92***	0.74***
Maharashtra	1.95***	1.28	2.10***	0.92	1.14**	1.93***	0.93
Madhya Pradesh	1.68***	1.22	2.57***	0.91	0.70**	2.06***	0.89
Bihar	2.17***	0.99	2.58***	0.70	0.65	2.28***	0.70**
Gujarat	1.23**	0.68***	2.74***	0.63***	0.79***	2.37***	0.62***
Assam	2.92***	0.80	2.69***	1.84**	0.61**	2.67***	1.55**
Odisha	3.10***	1.49	2.91***	1.19*	0.65***	2.80***	1.16*
West Bengal	4.17***	1.34	2.66***	1.08	0.77**	3.00***	1.07
Uttar Pradesh	1.62***	0.98	3.89***	1.00	1.01*	3.03***	0.92
Haryana	3.60***	1.61	3.48***	0.84	1.10*	3.48***	0.91
Delhi	2.22**	3.82**	3.75***	1.50*	1.48***	3.64***	1.60***
Punjab	4.59**	1.31*	4.30***	1.15	1.40***	4.40***	1.17
India	2.24	1.06	2.52	0.90	0.90	2.38	0.89

Note: States are arranged in ascending order of OOPE in public health centers. t-test shows significant differences in OOPE of Tamil Nadu with each of the state in respective category (*** *p* < 0.001, **p < 0.01, *p < 0.05)

### Inter-state variations on direct and indirect costs of hospitalization

Appendix 3 of Additional file 3 presents the state pattern of health spending segregated for direct medical cost (cost of medicines, tests, doctor’s fee, and bed charges) and indirect medical cost (transportation and others). The share of direct cost in public health centers in India was 76% and that of indirect cost was 24%. The state pattern is distinct. Among those using public health centers, the share of direct cost was the lowest in Tamil Nadu (23%) and highest in Uttar Pradesh and Punjab (87% each). Among poor using public health centers, the direct cost of hospitalization was also lowest in Tamil Nadu (14%) and the highest in Punjab (87%), followed by West Bengal (86%). The share of direct cost on hospitalization in public health centers among the poor in poorer states such as Bihar, Odisha, and Uttar Pradesh was over 70% of the total cost.

Table 5 presents the percentage distribution of medical expenditure on hospitalization by doctor’s fees, cost of medicines, cost of diagnostic tests, bed charges, other medical expenditure, transportation and other non-medical expenditure in public and private health centers, and poverty in a developed state, Tamil Nadu and a poorer state, Bihar. We have also presented the estimates of India. Among the poor using public health centers in India, the share of medicine to total health cost was 38, 13% on diagnostic tests, 7% on doctor’s fees, and 3% on bed charges. In the case of Bihar, the share of medicine on total cost of hospitalization was largest (45%) while it was least in Tamil Nadu (4%). The pattern was similar among the non-poor who were using public health centers. In public health centers of Tamil Nadu, the share of doctor’s fees, medicines, diagnostic tests, bed charges, and all other medical expenses accounted 0.47, 7.64, 7.72, 0.35 and 6.78% respectively. The expenditure on medicines accounted for the largest share of CHPE in all the states except Tamil Nadu. In public health centers in Bihar, the share of doctor’s fees, medicines, diagnostic tests, bed charges and all other medical expenses accounted for 12, 38, 9, 3 and 8% of the CHPE respectively. Similar state-wise variations were noticed in each of the component across the states of India.

**Table 5:** Share (%) of direct and indirect cost by poverty and type of health centers in India, Tamil Nadu, and Bihar, 2014

	Direct cost					Indirect cost	
Poverty	Doctor	Medicine	Diagnostic	Bed	Other medical	Transport	Other non-medical
India							
Poor using public health centers	6.69	37.75	13.59	2.75	9.62	10.52	19.08
Poor using private health centers	23.13	30.25	10.78	13.86	9.33	4.25	8.41
Non-poor using public health centers	7.97	33.60	12.31	3.12	14.81	9.29	18.91
Non-poor using private health centers	25.32	27.69	11.66	14.71	9.13	3.66	7.83
Utilization of public health centers (All)	7.60	34.79	12.68	3.01	13.31	9.65	18.96
Tamil Nadu							
Poor using public health centers	0.60	3.72	4.58	0.00	5.51	21.99	63.60
Poor using private health centers	32.63	22.80	10.00	14.88	7.36	3.93	8.41
Non-poor using public health centers	0.44	8.67	8.55	0.44	7.12	15.79	59.00
Non-poor using private health centers	28.65	25.73	11.34	13.55	9.13	3.64	7.96
Utilization of public health centers (All)	0.47	7.64	7.72	0.35	6.78	17.08	59.95
Bihar							
Poor using public health centers	6.69	45.48	9.99	3.17	7.82	9.98	16.86
Poor using private health centers	19.05	38.34	9.20	13.30	5.19	4.58	10.34
Non-poor using public health centers	15.29	33.29	9.20	3.33	9.35	10.64	18.91
Non-poor using private health centers	21.11	27.91	10.45	12.64	8.05	5.78	14.06
Utilization of public health centers (All)	12.07	37.85	9.50	3.27	8.87	10.39	18.15

**Fig. 4:**
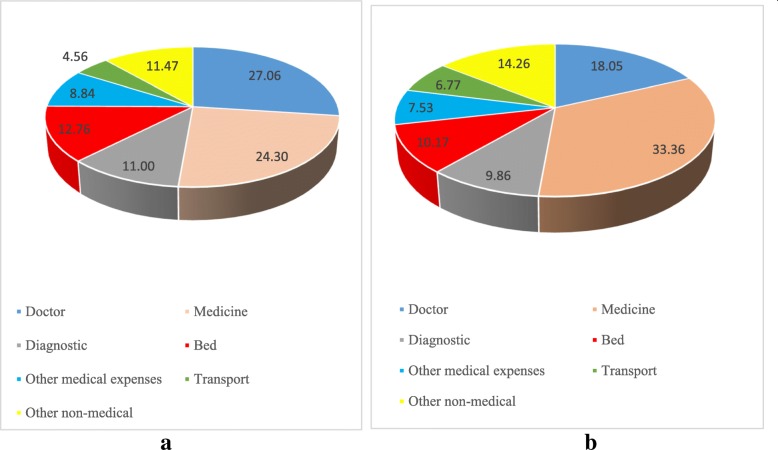
Share of hospitalization cost (%) by different components in (**a**) Tamil Nadu and (**b**) Bihar

Figure 4 presents the percentage share of direct and indirect cost, irrespective of poverty and type of health centers for Tamil Nadu and Bihar. In Tamil Nadu, the share of doctor’s fees, medicines, diagnostic tests, bed charges, and all other medical expenses was 27, 24, 11, 13, 9, 4, and 11%, while it was 33, 18, 10, 10, 7, 7, and 14% in Bihar. In general, differences on CHPE between richer and poorer state is large due to public health centers.

### Supply of healthcare services in public health centers in states of India

Table 6 presents the supply side factors that may have an impact on variations in CHPE across the states of India. In India, the shortfall of PHC from required norms was 17% and it was 29% for CHC. The shortfall of PHC was highest in Jharkhand (66%) while there was no shortfall of PHCs in Kerala, Chhattisgarh, Karnataka, and Assam. The shortfall of CHC was high in Bihar (91%), while there was no shortfall of CHC in Tamil Nadu and Kerala. Availability of bed per one lakh population is one of the most important supply side factors in any developing country. In India, the total number of beds per one lakh population was 114 in public hospitals and ranged from 137 in Delhi to 13 in Bihar. According to Indian public health standard (IPHS) norms, each PHC must have a qualified medical officer with at-least three nurses and six beds. The availability of human resources such as medical and paramedical staffs and infrastructure (availability of bed at PHC) was not satisfactory in many of the poorer states of India.

**Table 6:** Shortfall in public health facilities (%), primary health center, number of beds per one lakh population and health worker per 1000 population in States of India, 2013

States	Shortfall in public health facilities			Bed per one lakh population	Shortfall in primary health center			Distance between village to PHC	Health worker per 1000 population in 2011
	SC	PHC	CHC		No doctor	Less than 3 Nurse	less than 6 bed		
Tamil Nadu	0.0	1.8	0.0	86	2.2	31.6	61.5	7.9	4.3
Jharkhand	34.7	65.8	22.0	16	27.3	63.6	30.9	4.3	2.4
Telengana	15.4	16.7	29.2	NA	2.5	14.7	28.9	9.9	4.2
Andhra Pradesh	0.0	13.0	40.5	77	2.2	10.5	41.8	9.0	3.8
Rajasthan	0.0	13.5	7.3	56	14.8	43.9	23.4	11.0	3.0
Kerala	0.0	0.0	0.0	111	5.5	23.2	58.0	4.8	8.5
Chhattisgarh	0.0	0.0	18.7	42	52.2	96.3	13.2	8.8	2.3
Karnataka	0.0	0.0	42.3	85	10.6	22.1	8.3	7.0	4.3
Maharashtra	21.7	17.7	34.4	42	0.6	79.3	3.6	10.1	5.3
Madhya Pradesh	28.6	41.9	33.0	42	40.9	78.4	31.0	10.7	3.2
Bihar	47.8	39.2	91.0	13	5.6	51.8	14.9	8.3	1.8
Gujarat	9.2	10.2	1.2	59	NA	NA	NA	NA	4.1
Assam	21.2	0.0	53.8	33	6.1	59.4	70.1	6.4	4.2
Odisha	18.4	0.8	0.0	40	32.7	96.5	91.5	6.3	4.2
West Bengal	20.8	57.8	35.5	85	11.8	58.3	58.3	4.5	4.4
Uttar Pradesh	34.2	32.7	40.4	79	25.4	94.9	88.5	5.4	2.3
Haryana	23.5	17.8	19.7	31	13.8	28.9	48.8	6.9	3.8
Delhi	54.2	61.5	NA	137	NA	NA	NA	NA	7.8
Punjab	14.9	24.6	1.4	41	13.6	35.8	63.0	4.9	4.7
India	15.4	16.7	29.2	114	15.9	58.1	41.5	8.4	3.8

**Fig. 5:**
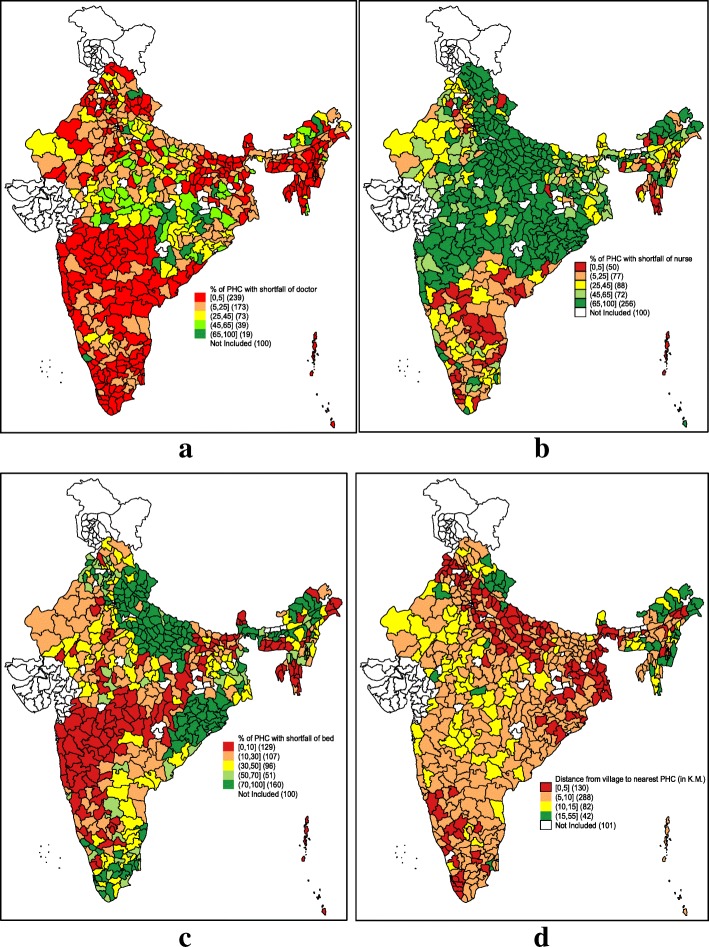
Geographical distribution of primary health center (PHC) (%) with shortfall of (**a**) doctor (**b**) shortfall of nurse (**c**) shortfall of bed (**d**) distance from village to PHC (in kilometer) in districts of India in 2013

Figure 5 presents the spatial distribution of districts with shortfall of human resources (doctors and nurses) and infrastructure (bed) in the PHC of India. Figure 5 (a) shows that shortfall of doctor in PHC in 131 districts of India was high (25% and above). The shortfall of nurses was high (65% and above) in the PHC of 256 districts in India (Fig. 5 b). Noticeably, all the districts of Odisha and Uttar Pradesh had shortfall of beds in PHC (Fig. 5 c). The average distance from the village to the nearest PHC was above 15 km (KM) in 45 districts, especially in the remote hilly areas of India (Fig. 5 d).

### Determinants of the cost of hospitalization

Table 7 presents the result of log-linear regression model that used CHPE as the dependent variable controlling for various socio economic and demographic factors. The model is estimated for India and two disparate states, namely, Bihar and Tamil Nadu. In India, all the socio-economic and demographic factors except place of residence adjusted in the model are significant. The composite variable of poverty and type of health centers are significant predictors in the model. The CHPE of poor using public health centers in India was 51% ((exp(0.71)-1)) lower than that of the non-poor using private health centers. Similarly, the CHPE of poor using private health centers was 18% ((exp(0.20)-1)) lower than that of the non-poor using private health centers. The CHPE of non-poor using public health centers was 43% ((exp(0.56)-1)) lower than that of the non-poor using private health centers.

**Table 7:** Result of log-linear regression model on cost of hospitalization in India, Bihar and Tamil Nadu

Parameters	Model-1 (India)	Model-2 (Bihar)	Model-3 (Tamil Nadu)
	Coef. 95% CI	Coef. 95% CI	Coef. 95% CI
Resident			
Rural®			
Urban	0.001 (−0.02, 0.03)	0.092(−0.02, 0.21)	0.041(− 0.04, 0.13)
Poverty and hospitalization			
Non-poor & using private health centers®			
Poor & using public health centers	−0.710***(− 0.75, − 0.67)	−0.683***(− 0.83, − 0.54)	−0.468**(− 0.85, − 0.09)
Poor & using private health centers	−0.197***(− 0.23, − 0.17	−0.280***(− 0.40, − 0.16)	− 0.245***(− 0.38, − 0.11)
Non-poor & using public health centers	−0.557***(− 0.59, − 0.52)	−0.473***(− 0.60, − 0.34)	−0.294(− 0.67, 0.08)
Age			
15–59®			
<=14	−0.075***(− 0.11, − 0.04)	− 0.015(− 0.15, 0.12)	0.056(− 0.08, 0.19)
60+	0.043**(0.01, 0.07)	0.080(− 0.07, 0.23)	− 0.029(− 0.13, 0.07)
Sex			
Male®			
Female	− 0.151***(− 0.17, − 0.13)	−0.256***(− 0.36, − 0.15)	−0.082(− 0.17, 0.00)
Religion			
Hindu®			
Muslim	−0.049**(− 0.08, − 0.01)	−0.084(− 0.23, 0.07)	0.054(− 0.09, 0.20)
Others	0.113***(0.07, 0.16)	0.189(− 0.23, 0.60)	− 0.020(− 0.18, 0.14)
Caste			
SC/ST®			
OBC/Other	0.083***(0.06, 0.11)	0.235(0.12, 0.35)	0.021(− 0.08, 0.12)
Surgery			
Not received®			
Free/partly free	0.449***(0.41, 0.49)	0.234**(0.04, 0.43)	0.469***(0.33, 0.61)
On payment	0.846***(0.82, 0.87)	0.696***(0.59, 0.80)	0.924***(0.83, 1.02)
Medicine			
Not received®			
Free/partly free	−0.011(− 0.20, 0.18)	0.320(− 0.23, 0.87)	0.013(− 0.84, 0.87)
On payment	0.793***(0.60, 0.98)	0.797**(0.25, 1.34)	1.386**(0.49, 2.28)
X-ray			
Not received®			
Free/partly free	0.365***(0.32, 0.41)	0.445(0.26, 0.63)	0.270(0.14, 0.40)
On payment	0.607***(0.58, 0.63)	0.550***(0.44, 0.66)	0.684***(0.57, 0.80)
Diagnostic			
Not received®			
Free/partly free	0.133***(0.09, 0.18)	0.041(− 0.12, 0.21)	0.118(− 0.06, 0.30)
On payment	0.632***(0.60, 0.66)	0.569***(0.45, 0.69)	0.529***(0.38, 0.68)
Insurance			
No health insurance®			
Not reimbursed	0.053***(0.03, 0.08)	−0.021(− 0.12, 0.08)	0.080(−0.08, 0.24)
Reimbursed	0.515***(0.46, 0.57)	0.485**(0.13, 0.84)	0.427***(0.19, 0.66)
Diseases			
Cancer®			
Bone disease	−0.843***(− 0.96,-0.73)	−1.360***(−1.85, − 0.87)	− 0.409(− 0.82, 0.00)
Diabetes	− 0.875***(− 1.00, − 0.75)	−1.629***(− 2.28, − 0.98)	− 0.402(− 0.82, 0.01)
Fever	−1.098***(− 1.20, − 0.99)	−1.804***(− 2.30, − 1.31)	− 0.667**(− 1.06, − 0.27)
High Blood Pressure	−1.086***(− 1.21, − 0.96)	−1.588***(− 2.21, − 0.97)	− 0.555**(− 1.08, − 0.03)
Accident	−0.899***(− 1.00, − 0.80)	−1.606***(− 2.07, − 1.15)	− 0.426**(− 0.82, − 0.04)
Jaundice	−0.681***(− 0.80, − 0.56)	− 1.472***(− 2.03, − 0.92)	0.120(− 0.34, 0.58)
Respiratory Diseases	−0.960***(− 1.07, − 0.85)	−1.592***(− 2.10, − 1.09)	− 0.487**(− 0.91, − 0.06)
Heart Diseases	−0.446(− 0.55, − 0.34)	−0.913***(− 1.42, 0.41)	0.043(− 0.36, 0.45)
Eye Diseases	− 1.664***(− 1.55, − 2.062)	− 2.062***(− 2.54, − 1.58)	−1.440***(− 1.83, − 1.05)
Tuberculosis	−0.683***(− 0.83, − 0.56)	−1.685***(− 2.22, − 1.16)	− 0.400(− 1.02, 0.22)
Blood disease	− 0.754***(− 0.88, − 0.62)	−1.158***(− 1.67, − 0.64)	− 0.328(− 0.84, 0.18)
Neurological	−0.609***(− 0.72, − 0.50)	−1.124***(− 1.63, − 0.62)	− 0.120(− 0.55, 0.31)
Others	−0.982***(− 1.08, − 0.88)	−1.641***(− 2.09, − 1.19)	− 0.293(− 0.68, 0.09)
Constant	8.446***(8.23, 8.66)	8.977***(8.25, 9.71)	7.390***(6.39, 8.39)

***p < 0.001, **p < 0.01, *p < 0.05.® Reference category. *CI* Confidence Interval

Among other variables, those who were hospitalized and underwent surgery, the CHPE had higher by 133% ((exp(0.85)-1)) than those hospitalized without surgery (Table 7). The results also revealed that those individuals were insured for health, spent 67% ((exp(0.51)-1)) higher CHPE compared to those who were not insured. Similarly, those individuals treated for any others NCDs had lower CHPE compared to those treated for cancer. The significant predictors in both Bihar and Tamil Nadu are poverty and type of hospital, surgery, medicine and diagnostic services, health insurance benefit and diseases. The general pattern in variable of interest (poverty and type of health centers) is similar in Bihar and Tamil Nadu but the coefficient varies.

### Robustness analysis

As a robustness check, we perform a tobit regression model using CHPE as depended variable. Appendix 4 of Additional file 4 presents the marginal effect along with 95% confidence interval of CHPE estimated for India, Bihar and Tamil Nadu. The CHPE of the individuals belonging to poor households and using public health centers for hospitalization in India was ₹4835 (*p* < 0.001, CI -5585, − 4085) lower than that of the non-poor who were using private health centers. Similarly, the CHPE of individuals belonging to poor households and using private health centers was ₹4585 (*p* < 0.001, CI -5314, − 3856), while the non-poor using public health centers spent ₹4921 (p < 0.001, CI -5782, − 4059) less than the non-poor using private health centers. The CHPE of individuals belonging to the poor households and using public health centers in Bihar was ₹4478 (*p* < 0.001, CI -8352, − 605), while poor households using private health centers spent ₹5983 (*p* < 0.001, CI -9334, − 2633), and non-poor households and using public health centers spent ₹3788 (p < 0.001, CI -8292, 716) less than the non-poor and using private health centers. Similarly, in Tamil Nadu CHPE of individuals belonging to poor households and using public health centers was ₹5482 (*p* < 0.001, CI -9302, − 1662), while that of poor households using private health centers was ₹5172 (p < 0.001, CI -7664, − 2681), individuals belonging to non-poor households using public health centers spent ₹5821 (*p* < 0.01, CI -9694, − 1990) less than the non-poor using private health centers. Our results of Tobit model supports the findings of the log-linear regression model.

## Discussion

The health spending in developing countries, unlike that in developed countries, is largely met by the households. Studies across developing countries suggests that poor households often resort to borrowing or selling assets to meet the OOPE for hospitalization [33, 40, 41, 57]. Many developing countries have introduced varying financial protection schemes and strengthened the public health centers to protect the poor and needy from high OOPE and rising health care costs. However, disparities in access and inequality in health outcome persists across and within countries. In India, rising health care cost is affecting the poor and vulnerable the most. Recent studies suggests that the state variations in CHS are large and the CHS has increased among the poor and marginalized [9, 27]. The public health centers in India are intended to provide free/affordable health services to the poor. But, the cost, and quality of services in public health centers vary largely across and within the states. Using the health survey data on hospitalization care, this study examined the inter-state variations of OOPE on hospitalization by level of poverty and type of health centers in India. The followings are the salient findings of the paper.

First, utilization of public health centers is higher among the poor than among the non-poor cutting across the states. More than half of the poor people in the poorer states of Assam, Odisha, Madhya Pradesh, Bihar, Jharkhand, and Chhattisgarh was hospitalized in public health centers, significantly higher than those in the developed states of Kerala and Andhra Pradesh. The findings suggest that poor people continued to rely on public health centers for utilization of inpatient services in India. Second, the OOPE accounts for over 95% of the cost of hospitalization of India. It varies from 85% in Delhi to 99% in the poorer states of Uttar Pradesh, Bihar and Jharkhand suggesting that most of the spending on hospitalization is largely met by the households in India. Third, the state pattern of OOPE in the public and private health centers is striking. The mean OOPE per episode of hospitalization in public health centers of Tamil Nadu was lowest among all states of India, while it was twice that of Tamil Nadu in the poorer states of Odisha, Assam and Bihar. We also found significant differences on OOPE in public health centers across the states of India. Though the CHPE was lowest in the public health centers of Tamil Nadu, it was not the case in private health centers. Many of the states had lower OOPE in private health centers than those in Tamil Nadu. The OOPE in private health centers was four times higher than that in public health centers in India. The state pattern suggests that CHPE in private health centers was highest in Delhi followed by Assam and lowest in Jharkhand followed by Gujarat.

Fourth, we found large variation in components of CHPE in India. For the poor using public health centers in India, the share of doctor’s fees account 7%, medicine 38%, diagnostic tests 14% and bed 3%. The proportion was much lower in Tamil Nadu than in Bihar. For example, in Tamil Nadu 4% of CHPE was spent on medicine compared to 45% in Bihar. Similarly, for the poor who used public health centers in Bihar, 7% of CHPE was spent on doctor’s fee, 10% spent on diagnostic tests and 3% on bed charge. In the case of Tamil Nadu, 1% of CHPE was spent on doctor’s fee and 5% spent on diagnostic tests. Fifth, the variations in the supply side factors such as availability of health facilities, doctors, paramedical, nurses, beds and medicine were large across the states of India. Doctor per 1000 population was the lowest in poorer states Bihar (1.8) and Uttar Pradesh (2.3) and highest in Kerala (8.5) [58]. Sixth, multivariate analysis suggests that the composite variables of poverty and utilization of health care centers were significant predictors of health spending in India. Poor people using public health centers in the state of Tamil Nadu spent less than those in Bihar, controlling for socio-economic correlates.

Our findings suggest large variations in utilization and OOPE on hospitalization among the poor in states of India. These findings are consistent with literature [47, 59–62]. We provide some plausible explanations. Public health centers continues to be the mainstay of health services for the poor while rich people preferring to use private health services [63, 64]. This is due to the higher ability to pay for health services among the non-poor, health system of the state and access to health insurance / reimbursement mechanism. Health is a state subject and the state government provide preventive, promotive and curative services. Studies have found that due to low quality of care, long waiting time, distance to health centers, and lack of availability of trained professionals in public health centers, people preferred to use private health centers in the poorer states of India [65, 66]. It may be mentioned that the central and state government has introduced various financial protection schemes for the poor and needy. The most prominent was Rashtriya Swasthya Bima Yojana (RSBY) introduced in 2008 specifically for the households living below the poverty line. Evaluative studies suggest that the RSBY has increased the utilization of public health services but the effect on OOPE was inclusive [17, 38, 43, 61, 67]. A number of schemes were launched by some states in India to provide health insurance to the poor and needy. These include the Chief Minister Comprehensive Health Insurance Schemes (CMCHIS) in Tamil Nadu, Rajiv Aarogyasri schemes in Andhra Pradesh and Telangana, Karunya health insurance schemes in Kerala, Mukhyamantri Amrutam in Gujarat, mahatma Jyotiba Phule Jan Arogya Joyana in Maharashtra, and Yashasvini and Vajpayee Arogyasree in Karnataka.

The CMCHIS schemes in Tamil Nadu are primarily intended for the poor to ensure that they get quality medical services for major ailments. These schemes covered costs up to ₹5 lacs including quality medical and surgical treatment in public and private health centers. Moreover, the relative allocation of state budget on health, and allocation within the state budget might be resulting in low OOPE for the public health centers users in Tamil Nadu. Besides publicly funded health insurance schemes, studies also suggest that good public health care infrastructure, distribution of free medicines, and health insurance for formal sector employees may be the other reasons for low OOPE in Tamil Nadu [68]. Similarly, in 2009, the Mukhya Mantri Jeevan Rakhsha Kosh was implemented even in the poorer state of Rajasthan that provided insurance coverage up to ₹5 lacs. Literature suggests that large inter-state variations in health spending [26, 61] was due to the limited effect of public funded health insurance (PFHI) schemes, particularly for the poor [43, 67, 69]. Furthermore, the shortfall of health infrastructure in public health centers, way below the prescribed norms in most of the poorer states in India, might be leading to high OOPE in poorer states. For instance, the number of beds per one lakh population is low in the poorer states like Bihar and high in developed states like Delhi and Kerala. There is evidence in developing country that the burden of health care increases with the increase in distance between home and health centers [7].

Recently, the Government of India has introduced the Ayushman Bharat, the largest ever health insurance scheme in the country. The program intended to provide financial protection to the bottom 40% of the population. The National Health Policy-2017 is also aimed to increase central government spending up to 2.5%, reduce medical impoverishment and reduce inequality in health spending by 2025. However, the effect of Ayushman Bharat could not be assessed due to data limitations. Second, data on health infrastructure were not collected in the NSS survey and could not be analyzed. Establishing from availability of health infrastructure from other sources could not be carried out due to time constraint.

## Conclusion

This study concludes that the poor people in poorer states have not benefited adequately from public health care services and are not protected against unanticipated health care costs. Low public health investment, poor public health infrastructures, non-availability of medicines and diagnosis tests and user fees are the main reasons for the high inter-state variations of OOPE in India. Increasing public spending, effective management of public health centers, effective implementation of state sponsored health insurance schemes, provisioning of medicines and diagnostic test at public health centers of poorer states can reduce inter-state variations of OOPE in India. Some of the best practices of health system within the country, as illustrated by Tamil Nadu, may be adopted in the poor performing states to reduce inter-state variations in health spending among the poor.

## Additional files

Additional file 1: **Appendix 1.** State variations in hospitalization (%) in public and private health centers by broad disease in India, 2014 (docx 15 kb). (DOCX 14 kb)

Additional file 2: **Appendix 2.** Mean out-of-pocket expenditure on cost of hospitalization (₹) by broad disease in public and private health centers of India, 2014 (docx 17 kb). (DOCX 15 kb)

Additional file 3: **Appendix 3**. Percentage share of direct and indirect cost of hospitalization by poverty and type of health care centers among major states in India, 2014 (docx 16 kb). (DOCX 16 kb)

Additional file 4: **Appendix 4.** Marginal effect and 95% Confidence Interval (CI) of hospitalization cost per episode in India, Bihar and Tamil Nadu, 2014. (DOCX 15 kb)

## Data Availability

NSS data used in this study for analysis is publicly available and unit level data could be obtained upon a data request through http://www.icssrdataservice.in/datarepository/index.php/catalog/107, subject to non-profit and academic interest only. DLHS-4 data used in this study is also publicly available and unit level data could be accessed on a data request from http://rchiips.org/DLHS-4.html. Availability of public health institutions, bed per 1 lacs population, health worker per 1000 population taken from Family Welfare Statistics in India through https://mohfw.gov.in/documents/staistics which is publicly available for all. In another case, the corresponding author of this paper may contacted for data.

## Abbreviations

CDs: Communicable diseases
CHC: Community health center
CHPE: Cost of hospitalization per episode
CHS: Catastrophic health spending
CI: Confidence Interval
CMCHIS: Chief minister comprehensive health insurance schemes
DH: District hospital
DLHS: District level households and facility survey
EAGs: Empowered action group states
Exp: exponentiation
GDP: Gross domestic product
ICSSR: Indian council of social science research
IIPS: International institute for population sciences
IPHS: Indian public health standard
MoHFW: Ministry of health and family welfare
MPCE: Monthly per capita expenditure
NCDs: Non-communicable diseases
NSS: National sample survey
NSSO: National sample survey organization
OBC: Other backward classes
OOPE: Out-of-pocket expenditure
PFHI: Publicly funded health insurance
PHC: Primary health center
RSBY: Rashtriya swasthya bima yojana
SC: Sub center
SC/ST: Schedule caste/Schedule tribe
SDGs: Sustainable development goals
UHC: Universal health coverage
