# Supplementary material for: Do poor people in the poorer states pay more for healthcare in India?
Source: BMC Public Health. 2019 Jul 30;19:1020. doi: 10.1186/s12889-019-7342-8 (PMC6668144; doi:10.1186/s12889-019-7342-8)
Supplement: Supplementary file 1 — Appendix 1. State variations in hospitalization (%) in public and private health centers by broad disease in India, 2014 (docx 15 kb). (DOCX 14 kb) [file 12889_2019_7342_MOESM1_ESM.docx]

**Appendix 1** State variations in hospitalization (%) in public and private health centres by broad disease in India, 2014

| States | Public health centres | | | | | Private health centres | | | | |
| --- | --- | --- | --- | --- | --- | --- | --- | --- | --- | --- |
|  | Non-communicable diseases | Communicable diseases & maternity | Accident | Others | Number of cases | Non-communicable diseases | Communicable diseases & maternity | Accident | Others | Number of cases |
| Tamil Nadu | 57.30 | 30.04 | 9.99 | 2.66 | 1559 | 75.08 | 13.74 | 8.59 | 2.58 | 2230 |
| Jharkhand | 21.31 | 71.03 | 6.20 | 1.45 | 552 | 62.84 | 28.45 | 6.80 | 1.91 | 638 |
| Telangana | 59.60 | 27.31 | 12.11 | 0.98 | 336 | 61.71 | 24.37 | 12.20 | 1.71 | 939 |
| Andhra Pradesh | 49.66 | 35.96 | 11.88 | 2.50 | 586 | 70.00 | 18.62 | 8.97 | 2.41 | 1803 |
| Rajasthan | 40.68 | 50.76 | 7.74 | 0.82 | 1551 | 61.38 | 26.81 | 9.89 | 1.93 | 1079 |
| Kerala | 67.73 | 13.87 | 12.52 | 5.88 | 981 | 73.90 | 13.28 | 6.50 | 6.31 | 2021 |
| Chhattisgarh | 45.18 | 42.87 | 5.48 | 6.48 | 471 | 73.88 | 16.86 | 6.37 | 2.89 | 429 |
| Karnataka | 43.82 | 48.25 | 5.46 | 2.47 | 838 | 72.47 | 18.38 | 6.51 | 2.64 | 1994 |
| Maharashtra | 45.22 | 47.25 | 5.07 | 2.46 | 1320 | 66.41 | 20.77 | 9.36 | 3.45 | 3689 |
| Madhya Pradesh | 38.68 | 54.60 | 4.14 | 2.59 | 1726 | 68.43 | 18.38 | 9.35 | 3.84 | 1386 |
| Bihar | 23.01 | 69.80 | 5.89 | 1.29 | 1092 | 53.52 | 31.34 | 11.37 | 3.78 | 1283 |
| Gujarat | 54.42 | 39.05 | 6.16 | 0.37 | 699 | 62.92 | 29.28 | 7.01 | 0.79 | 2000 |
| Assam | 33.76 | 57.86 | 4.81 | 3.57 | 1395 | 45.52 | 34.84 | 5.42 | 14.23 | 315 |
| Odisha | 52.73 | 37.00 | 8.39 | 1.88 | 1608 | 65.95 | 20.56 | 10.07 | 3.42 | 504 |
| West Bengal | 54.32 | 31.07 | 11.05 | 3.56 | 2918 | 63.93 | 23.00 | 7.11 | 5.95 | 1573 |
| Uttar Pradesh | 28.82 | 64.25 | 5.73 | 1.20 | 2336 | 56.43 | 30.05 | 8.38 | 5.14 | 4103 |
| Haryana | 39.03 | 49.74 | 5.82 | 5.41 | 466 | 61.58 | 29.65 | 5.35 | 3.42 | 807 |
| Delhi | 58.90 | 36.93 | 3.49 | 0.68 | 402 | 66.22 | 20.59 | 4.70 | 8.48 | 468 |
| Punjab | 51.94 | 38.18 | 7.63 | 2.25 | 389 | 66.80 | 21.79 | 8.72 | 2.70 | 939 |
| India | 45.18 | 44.66 | 7.69 | 2.47 | 27439 | 65.82 | 22.19 | 8.39 | 3.60 | 30017 |
