# Supplementary material for: Do poor people in the poorer states pay more for healthcare in India?
Source: BMC Public Health. 2019 Jul 30;19:1020. doi: 10.1186/s12889-019-7342-8 (PMC6668144; doi:10.1186/s12889-019-7342-8)
Supplement: Supplementary file 2 — Appendix 2. Mean out-of-pocket expenditure on cost of hospitalization (₹) by broad disease in public and private health centers of India, 2014 (docx 17 kb). (DOCX 15 kb) [file 12889_2019_7342_MOESM2_ESM.docx]

**Appendix 2** Mean out-of-pocket expenditure on cost of hospitalization (₹) by broad disease in public and private health centers of India, 2014

| States | Public health centers | | | | Private health centers | | | | Combined | | | |
| --- | --- | --- | --- | --- | --- | --- | --- | --- | --- | --- | --- | --- |
|  | Non-communicable diseases | Communicable diseases & maternity | Accident | Others | Non-communicable diseases | Communicable diseases & maternity | Accident | Others | Non-communicable diseases | Communicable diseases & maternity | Accident | Others |
| Tamil Nadu | 2187 | 2327 | 3651 | 2913 | 26008 | 29766 | 44139 | 25102 | 18226 | 13808 | 26933 | 16315 |
| Jharkhand | 6696 | 2823 | 7364 | 893 | 16364 | 14649 | 16004 | 12621 | 13588 | 5806 | 11511 | 7049 |
| Telengana | 4445 | 3000 | 4833 | 4981 | 26894 | 23356 | 31598 | 23592 | 20985 | 17391 | 24412 | 20339 |
| Andhra Pradesh | 5254 | 3133 | 4361 | 1696 | 25404 | 16030 | 27227 | 17677 | 21411 | 10844 | 20008 | 13436 |
| Rajasthan | 6503 | 1938 | 7977 | 6392 | 25402 | 15476 | 33437 | 54982 | 15260 | 5081 | 18734 | 34215 |
| Kerala | 4249 | 3096 | 6419 | 3836 | 25090 | 20423 | 34107 | 13730 | 18436 | 14387 | 20367 | 10535 |
| Chhattisgarh | 5808 | 3281 | 4402 | 3492 | 22619 | 17486 | 52522 | 21502 | 15697 | 6914 | 28634 | 8548 |
| Karnataka | 5349 | 3579 | 6888 | 6198 | 18584 | 19866 | 38833 | 42477 | 15632 | 10829 | 29738 | 31313 |
| Maharashtra | 5760 | 2779 | 11585 | 4270 | 26000 | 19741 | 40935 | 28240 | 22115 | 12236 | 36274 | 23470 |
| Madhya Pradesh | 8827 | 1996 | 7812 | 4300 | 25183 | 16943 | 36386 | 31494 | 17385 | 4578 | 24484 | 17340 |
| Bihar | 10796 | 3436 | 8825 | 4943 | 19090 | 17547 | 23064 | 32071 | 16203 | 7182 | 17485 | 23995 |
| Gujarat | 8524 | 1427 | 7649 | 4615 | 17712 | 11729 | 35921 | 20291 | 15552 | 8417 | 29196 | 18036 |
| Assam | 7840 | 5101 | 7497 | 12339 | 31475 | 19236 | 21934 | 148788 | 11691 | 6231 | 9516 | 62205 |
| Odisha | 6589 | 4800 | 15833 | 6180 | 31909 | 19573 | 62600 | 31968 | 12586 | 6590 | 26570 | 14193 |
| West Bengal | 7180 | 3241 | 9186 | 35539 | 30544 | 19433 | 55145 | 37612 | 14910 | 7082 | 18971 | 36395 |
| Uttar Pradesh | 16406 | 2347 | 10445 | 35526 | 25678 | 19608 | 37832 | 45679 | 23272 | 9344 | 29089 | 44276 |
| Haryana | 13816 | 2579 | 14266 | 15491 | 27821 | 21345 | 32311 | 13105 | 24427 | 12742 | 25922 | 14165 |
| Delhi | 11352 | 4741 | 6466 | 7335 | 47187 | 31858 | 98775 | 30001 | 30292 | 14431 | 59388 | 28309 |
| Punjab | 14275 | 4135 | 19017 | 4274 | 36426 | 20120 | 42354 | 20733 | 29990 | 12447 | 34988 | 15705 |
| India | 7485 | 2975 | 8624 | 12722 | 24927 | 19106 | 37304 | 31839 | 18587 | 9007 | 24905 | 24903 |
