# Supplementary material for: Do poor people in the poorer states pay more for healthcare in India?
Source: BMC Public Health. 2019 Jul 30;19:1020. doi: 10.1186/s12889-019-7342-8 (PMC6668144; doi:10.1186/s12889-019-7342-8)
Supplement: Supplementary file 3 — Appendix 3. Percentage share of direct and indirect cost of hospitalization by poverty and type of health care centers among major states in India, 2014 (docx 16 kb). (DOCX 16 kb) [file 12889_2019_7342_MOESM3_ESM.docx]

**Appendix 3** Percentage share of direct and indirect cost of hospitalization by poverty and type of health center among major states in India, 2014

| States | Poor using public health centers | | Poor using private health centers | | Non-poor using public health centers | | Non-poor using private health centers | | All | | Public health centers | | Private health centers | |
| --- | --- | --- | --- | --- | --- | --- | --- | --- | --- | --- | --- | --- | --- | --- |
|  | Direct | Indirect | Direct | Indirect | Direct | Indirect | Direct | Indirect | Direct | Indirect | Direct | Indirect | Direct | Indirect |
| Tamil Nadu | 14.41 | 85.59 | 90.36 | 9.64 | 25.76 | 74.24 | 91.87 | 8.13 | 88.45 | 11.55 | 23.42 | 76.58 | 91.76 | 8.24 |
| Jharkhand | 65.37 | 34.63 | 87.57 | 12.43 | 74.72 | 25.28 | 85.09 | 14.91 | 82.68 | 17.32 | 72.06 | 27.94 | 85.78 | 14.22 |
| Telangana | 57.87 | 42.13 | 91.51 | 8.49 | 65.01 | 34.99 | 92.41 | 7.59 | 90.72 | 9.28 | 63.61 | 36.39 | 92.29 | 7.71 |
| Andhra Pradesh | 63.77 | 36.23 | 88.85 | 11.15 | 62.62 | 37.38 | 90.63 | 9.37 | 88.78 | 11.22 | 62.77 | 37.23 | 90.55 | 9.45 |
| Rajasthan | 54.34 | 45.66 | 84.97 | 15.03 | 69.65 | 30.35 | 88.77 | 11.23 | 83.15 | 16.85 | 66.08 | 33.92 | 88.43 | 11.57 |
| Kerala | 64.48 | 35.52 | 92.31 | 7.69 | 62.59 | 37.41 | 92.90 | 7.10 | 90.40 | 9.60 | 62.77 | 37.23 | 92.88 | 7.12 |
| Chhattisgarh | 65.97 | 34.03 | 91.44 | 8.56 | 70.58 | 29.42 | 91.09 | 8.91 | 87.19 | 12.81 | 69.01 | 30.99 | 91.2 | 8.8 |
| Karnataka | 70.86 | 29.14 | 89.01 | 10.99 | 69.41 | 30.59 | 90.73 | 9.27 | 88.59 | 11.41 | 69.87 | 30.13 | 90.4 | 9.6 |
| Maharashtra | 75.00 | 25.00 | 92.74 | 7.26 | 76.76 | 23.24 | 93.49 | 6.51 | 92.45 | 7.55 | 76.11 | 23.89 | 93.38 | 6.62 |
| Madhya Pradesh | 62.66 | 37.34 | 89.05 | 10.95 | 79.97 | 20.03 | 90.32 | 9.68 | 86.58 | 13.42 | 74.49 | 25.51 | 90.01 | 9.99 |
| Bihar | 75.93 | 24.07 | 87.30 | 12.70 | 77.25 | 22.75 | 86.13 | 13.87 | 83.93 | 16.07 | 76.8 | 23.2 | 86.49 | 13.51 |
| Gujarat | 71.26 | 28.74 | 91.58 | 8.42 | 86.66 | 13.34 | 93.84 | 6.16 | 92.71 | 7.29 | 84.86 | 15.14 | 93.57 | 6.43 |
| Assam | 76.83 | 23.17 | 88.52 | 11.48 | 79.69 | 20.31 | 89.79 | 10.21 | 84.27 | 15.73 | 78.76 | 21.24 | 89.66 | 10.34 |
| Odisha | 69.84 | 30.16 | 84.92 | 15.08 | 75.32 | 24.68 | 89.03 | 10.97 | 81.28 | 18.72 | 72.97 | 27.03 | 88.12 | 11.88 |
| West Bengal | 85.66 | 14.34 | 91.67 | 8.33 | 83.00 | 17.00 | 91.84 | 8.16 | 89.14 | 10.86 | 83.92 | 16.08 | 91.82 | 8.18 |
| Uttar Pradesh | 77.76 | 22.24 | 91.49 | 8.51 | 88.61 | 11.39 | 92.91 | 7.09 | 91.71 | 8.29 | 86.8 | 13.2 | 92.65 | 7.35 |
| Haryana | 79.01 | 20.99 | 92.68 | 7.32 | 79.33 | 20.67 | 90.84 | 9.16 | 89.66 | 10.34 | 79.26 | 20.74 | 91.26 | 8.74 |
| Delhi | 69.07 | 30.93 | 93.14 | 6.86 | 82.26 | 17.74 | 95.41 | 4.59 | 93.17 | 6.83 | 81.26 | 18.74 | 95.32 | 4.68 |
| Punjab | 86.74 | 13.26 | 93.33 | 6.67 | 87.16 | 12.84 | 93.91 | 6.09 | 92.91 | 7.09 | 87.08 | 12.92 | 93.87 | 6.13 |
| India | 72.77 | 27.23 | 90.51 | 9.49 | 77.01 | 22.99 | 91.92 | 8.08 | 89.27 | 10.73 | 75.89 | 24.11 | 91.72 | 8.28 |
