# Supplementary material for: Do poor people in the poorer states pay more for healthcare in India?
Source: BMC Public Health. 2019 Jul 30;19:1020. doi: 10.1186/s12889-019-7342-8 (PMC6668144; doi:10.1186/s12889-019-7342-8)
Supplement: Supplementary file 4 — Appendix 4. Marginal effect and 95% Confidence Interval (CI) of hospitalization cost per episode in India, Bihar and Tamil Nadu, 2014. (DOCX 15 kb) [file 12889_2019_7342_MOESM4_ESM.docx]

**Appendix 4:** Marginal effect and 95% Confidence Interval (CI) of hospitalization cost per episode in India, Bihar and Tamil Nadu, 2014.

| Parameters | Reference categories | India | Bihar | Tamil Nadu |
| --- | --- | --- | --- | --- |
| Resident | Rural |  |  |  |
| Urban |  | 1843***(1234, 2451) | 4147**(1306, 6989) | 2657**(702, 4613) |
| Poverty and hospitalization | Non-poor & using private health services |  |  |  |
| Poor & using public health centers |  | -4835***(-5585, -4085) | -4478**(-8352, -605) | -5482**(-9302, -1662) |
| Poor & using private health centers |  | -4585***(-5314, -3856) | -5983***(-9334, -2633) | -5172***(-7664, -2681) |
| Non-poor & using public health centers |  | -4921***(-5782, -4059) | -3788(-8292, 716) | -5842**(-9694, -1990) |
| Age | 15-59 |  |  |  |
| <=14 |  | -1602***(-2240, -965) | -1446(-4160, 1268) | -1043(-3149, -3149) |
| 60+ |  | 1757***(813, 2700) | -296(-4647, 4055) | 1177(-1814, 4168) |
| Sex |  |  |  |  |
| Female | Male | -3549***(-4221, -2876) | -4859**(-7615, -2102 | -2215**(-4351, -80) |
| Religion | Hindu |  |  |  |
| Muslim |  | -1344***(-1994, -694) | -1536(-3639, 567) | 1178(-4509, 6866) |
| Others |  | 1304(-22, 2630) | -8797**(-16377, -1216) | 1139(-2056, 4333) |
| Caste | SC/ST |  |  |  |
| OBC/Other |  | 1739***(1263, 2214) | 3459***(1609, 5308) | -175(-1582, 1232) |
| Surgery | Not received |  |  |  |
| Free/partly free |  | 3403***(2688, 4118) | 3272(-218, 6763) | 4607***(3174, 6040) |
| On payment |  | 17899***(16753, 19045) | 11637***(7994, 15280) | 22287***(17801, 26774) |
| Medicine | Not received |  |  |  |
| Free/partly free |  | 56(-1933, 2046 | 1239(-2531, 5009) | -23(-5845, 5800) |
| On payment |  | 1428(-553, 3408) | 483(-3666, 4631) | -566(-6929, 5797) |
| X-ray | Not received |  |  |  |
| Free/partly free |  | -73(-427, 281) | -14(-2149, 2122) | 662(-528, 1852) |
| On payment |  | 5157***(4723, 5592) | 5092***(3232, 6953) | 6870***(5147, 8594) |
| Diagnostic | Not received |  |  |  |
| Free/partly free |  | 1828***(1468, 2188) | 495(-1292, 2282) | 627(-1040, 2294) |
| On payment |  | 3749***(3323, 4174) | 3454***(1425, 5483) | 4641***(2454, 6829) |
| Insurance | No insurance |  |  |  |
| No insurance benefit |  | 105(-522, 732) | -763(-3232, 1706) | 5609(-1337, 12554) |
| Insurance benefited |  | 13931***(10021, 17840) | 38514(-4447, 81476) | 12173**(2158, 22188) |
| Diseases | Cancer |  |  |  |
| Bone disease |  | -34211***(-42624, -25798) | -40626***(-64809, -16444) | -24072(-52402, 4258) |
| Diabetes |  | -37531***(-45797, -29265) | -45751***(-70328, -21174) | -26198(-52972, 576) |
| Fever |  | -38002***(-46243, -29761) | -45664***(-69665, -21663) | -25032(-52179, 2115) |
| High BP |  | -35278***(-43750, -26806) | -41237***(-67825, -14650) | -18971(-46984, 9041) |
| Accident |  | -34749***(-43142, -26355) | -44176***(-67848, -20503) | -23883(-51491, 3724) |
| Jaundice |  | -34187***(-42707, -25666) | -42411***(-66821, -18001) | -20352(-47816, 7111) |
| Respiratory |  | -36952***(-45179, -28726) | -44059***(-67822, -20297) | -23770(-50893, 3353) |
| Heart |  | -21690***(-30334, -13046) | -15605(-49096, 17885) | -7284(-36574, 22006) |
| Eye |  | -46720***(-54936, -38503) | -51971***(-75497, -28445) | -35859***(-63351, -8368) |
| Tuberculosis |  | -34931***(-43237, -26624) | -46980***(-70867, -23093) | -18573(-46509, 9362) |
| Blood disease |  | -34091***(-43035, -25148) | -40650***(-64289, -17011) | -18204(-47140, 10731) |
| Neurological |  | -31628***(-40015, -23242) | -34901***(-59371, -10432) | -14157(-43922, 15608) |
| Others |  | -38408***(-46684, -30131) | -45216***(-68799, -21634) | -26040(-54062, 1981) |

***p<0.001, **p<0.01, *p<0.05.
